# Quaternion-Based Signal Analysis for Motor Imagery Classification from Electroencephalographic Signals

**DOI:** 10.3390/s16030336

**Published:** 2016-03-05

**Authors:** Patricia Batres-Mendoza, Carlos R. Montoro-Sanjose, Erick I. Guerra-Hernandez, Dora L. Almanza-Ojeda, Horacio Rostro-Gonzalez, Rene J. Romero-Troncoso, Mario A. Ibarra-Manzano

**Affiliations:** 1 Laboratorio de Sistemas Bioinspirados, Departamento de Ingeniería Electrónica, DICIS, Universidad de Guanajuato, Carr. Salamanca-Valle de Santiago KM. 3.5 + 1.8 Km., Salamanca 36885, Mexico; p.batresmendoza@ugto.mx (P.B.-M.); ei.guerrahernandez@ugto.mx (E.I.G.-H.); hrostrog@ugto.mx (H.R.-G.); 2 Departamento de Arte y Empresa, DICIS, Universidad de Guanajuato, Carr. Salamanca-Valle de Santiago KM. 3.5 + 1.8 Km., Salamanca 36885, Mexico; cmontoro@ugto.mx; 3 Cuerpo Académico de Telemática, DICIS, Universidad de Guanajuato, Carr. Salamanca-Valle de Santiago KM. 3.5 + 1.8 Km., Salamanca 36885, Mexico; troncoso@hspdigital.org (R.J.R.-T.); ibarram@ugto.mx (M.A.I.-M.); 4 Laboratorio de Procesamiento Digital de Señales, Departamento de Ingeniería Electrónica, DICIS, Universidad de Guanajuato, Carr. Salamanca-Valle de Santiago KM. 3.5 + 1.8 Km., Salamanca 36885, Mexico; 5 Departamento de Ingeniería Electrónica, DICIS, Universidad de Guanajuato, Carr. Salamanca-Valle de Santiago KM. 3.5 + 1.8 Km., Salamanca 36885, Mexico

**Keywords:** quaternion-based signal analysis (QSA), electroencephalography (EEG), motor imagery, brain-computer interface (BCI)

## Abstract

Quaternions can be used as an alternative to model the fundamental patterns of electroencephalographic (EEG) signals in the time domain. Thus, this article presents a new quaternion-based technique known as quaternion-based signal analysis (QSA) to represent EEG signals obtained using a brain-computer interface (BCI) device to detect and interpret cognitive activity. This quaternion-based signal analysis technique can extract features to represent brain activity related to motor imagery accurately in various mental states. Experimental tests in which users where shown visual graphical cues related to left and right movements were used to collect BCI-recorded signals. These signals were then classified using decision trees (DT), support vector machine (SVM) and k-nearest neighbor (KNN) techniques. The quantitative analysis of the classifiers demonstrates that this technique can be used as an alternative in the EEG-signal modeling phase to identify mental states.

## 1. Introduction

The interest in establishing direct communication between the brain and other external devices using electroencephalographic (EEG) signals has increased with the use of brain-computer interface (BCI) systems. According to Wolpaw [1], “BCI interfaces allow to record the brain signals of an individual, extract their characteristics and turn them into artificial outputs that operate outside or in your own body”. In other words, the interface establishes a channel of communication and control between an individual and an external device. BCI systems use EEG activity to perform various tasks, such as controlling cursor movements [2], browsing the web [3,4], selecting letters or icons [5,6,7,8], communicating with virtual environments (e.g., in games) [9,10], robot navigation [11,12,13], controlling a wheelchair [14,15,16,17] or operating prosthetics [18,19,20], among others.

By nature, EEG signals are non-linear and non-stationary. Usually, these signals are processed and analyzed using various mathematical methods to gather information regarding the frequency components and, in turn, the functional relationships between brain areas. The most-commonly used techniques in the analysis are the fast Fourier transform (FFT), power spectral density (PSD), Hjorth parameters and the discrete wavelet transform (DWT). Several strategies have been presented to analyze brain signals by extracting and classifying EEG signals for cognitive-movement detection purposes. For instance, Hongyu [21] presents an on-line classification method for BCI based on common spatial patterns (CSP) for feature extraction, using support vector machine (SVM) as a classifier for imagined hand and foot movements achieving accuracy results of 86.3%, 91.8% and 92.0% with three subjects. Similarly, Bhattacharyya [22] conducted a study of comparative performance analysis of different classifiers, linear discriminant analysis (LDA), quadratic discriminant analysis (QDA), k-nearest neighbor (KNN), linear SVM, radial basis function (RBF) SVM and naïve Bayesian) to differentiate EEG signals for left-right limb movement, with SVM being the most accurate at 82.14%. In turn, Jiralerspong [23] conducted an experimental test of three mental states using FFT with a hamming window function that resulted in a 72% recognition rate. These strategies show good accuracy rates, but the information is extracted within a frequency or time-frequency domain, thereby losing vital information. On the other hand, some studies have been conducted based on multi-scale entropy (MSE) analysis to detect features of biological signals, as shown in [24], where Costa, *et al.* present a generalization of multi-scale entropy to analyze the structure of time series of heartbeats. Similarly, Mossabber [25] uses multivariate multi-scale entropy (MMSE) as a generalization of MSE to be adapted for biological and physical systems. Morabito, *et al.* [26] also use a multivariate multi-scale methodology to assess the complexity of physiological systems by means of permutation entropy whereby time series are processed in segments. However, the analysis does not appear to analyze several signals at the same time, and the performance of signals is based on patterns, which results in magnitude being weighted differently.

An alternative would be to conduct the signal analysis within a time domain. Quaternions can be an alternative to model EEG signals because they provide us with a mathematical notation to represent object orientations and rotations three-dimensionally, which makes it possible to represent EEG multichannel signals beyond what traditional methods allow. Recent studies have used quaternion algebra with traditional methods. For instance, Furman [27] uses the so-called quaternion Fourier transform (QFT) to process 3D images, and claims that the rotation operation is faster than when using a matrix-based method. In addition, Zhao [28] uses quaternion principle component analysis (QPCA) to represent EEG multichannel epilepsy signals with better results than those obtained using a traditional approach.

There are some considerable advantages in using quaternions, and thus a new technique to represent EEG signals visually using quaternion algebra to classify brain activity in relation to visual cues is presented here. The visual representation, extraction and classification of EEG features correspond to the cognitive activity recorded as the brain processes motor images. Motor imagery can be defined as a mental process linked to a motor action without any overt motor output [29]. In the case of this study, three kinds of visual cues were used: left, right and waiting time. As a result, EEG signals gathered using the BCI device were divided into blocks that contained information from only four of the 14 sensors. Each block was represented using quaternions to extract the signal features, and were later classified (off-line) using three techniques: KNN, SVM and decision trees (DT). Finally, the various classification results obtained when detecting classes were used to validate the performance levels.

This paper is organized as follows: Section 2 is an introduction to quaternions, BCI systems and classifiers; Section 3 includes a description the QSA technique; Section 4 accounts for the experimental tests; Section 5 features a discussion of the QSA implementation results; and Section 6 ends with some conclusions.

## 2. Preliminaries

This section provides a brief but accurate description of the key elements used to develop the study that is, quaternion algebra and the BCI device used to acquire the brain signals.

### 2.1. Quaternions

Quaternions were proposed in 1843 by Hamilton [30], as a set of four constituents (one real component and three imaginary) of the form: *q = w + ix + jy + kz*, where *w*, *x*, *y*, *z* ∈ ℝ and *i*, *j*, *k* are symbols of three imaginary quantities known as imaginary units. These units follow these rules:

i^2^ = j^2^ = k^2^ = ijk = −1 (1)

ij = k, jk = i, ki = j

ji = −k, kj = −i, ik = −j

A quaternion can be described as:

q = (*s* + **v**), **v** = (x, y, z) (2)

where *s* and **v** are known as the quaternion’s scalar and vector, respectively. When *s* = 0, *q* is known as a pure quaternion.

Table 1 summarizes the basic operations of quaternion algebra, where *q* and *p* are two quaternions, whilst the dot and the cross represent the usual scalar and vector products.

Note that the multiplication of quaternions is a not a commutative operation; instead, it is associative and distributive in relation to the addition.

Quaternions with a norm equal to one are known as unit (or normalized) quaternions. If *q* is a unit quaternion, it can be written as [31]:

(3) q=cosθ+sinθ e,‖e‖=1

where cosθ=s, sinθ=‖v‖ and u=v/‖v‖. Equally, the previous equation can be used to represent vector rotations.

(4) $$q=\cos\theta +\sin\theta \;e=\;ba^{-1}$$

where **a** and **b** are any two vectors having the same length, the angle between **a** and **b** is *θ,* **e** is perpendicular to both **a** and **b**, and **a**, **b**, and **e** form a right-handed set.

For a rotation of angle *θ* around a unit vector **a**, *q* must be formed thus:

(5) $$q=\cos\left(\frac{\theta }{2}\right)+a\;\sin\left(\frac{\theta }{2}\right)$$

Furthermore, the operation to be performed on a vector **r** to produce a rotated vector **r**’ is:

(6) $$r^{\prime }=qrq^{-1}=\left(\cos\left(\frac{\theta }{2}\right)+\;a\;\sin\left(\frac{\theta }{2}\right)\right)r\left(\cos\left(\frac{\theta }{2}\right)-\;a\;\sin\left(\frac{\theta }{2}\right)\right)$$

Equation (6) is a useful representation that makes the rotation of a vector easier. We can see that **r** is the original vector, ***r****’* is the rotated quaternion, and *q* is the quaternion that defines the rotation.

### 2.2. BCI System

**Figure 1:**
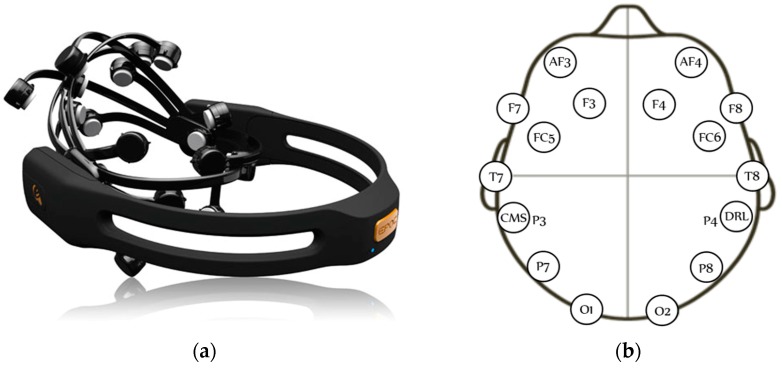
BCI System: (**a**) Emotiv Epoc headset; and (**b**) Emotiv Epoc electrode arrangement.

BCI devices capture brain signals of individuals that will be subsequently processed and analyzed. Depending on how signals are captured, BCI devices are classified as invasive and non-invasive. The Emotiv Epoc headset (Figure 1a) is a non-invasive mobile BCI device with a gyroscopic sensor and 14 EEG channels (electrodes) and two reference channels (CMS/DRL with a 128 Hz sample frequency). The distribution of sensors in the headset is based on the international 10–20 electrode placement system with two sensors as reference for proper placement on the head. This device obtains the brain activity of an individual, and is able to detect and process their thoughts, feelings and expressions in real time. Based on the 10–20 system, the channels are: AF3, F7, F3, FC5, T7, P7, O1, O2, P8, T8, FC6, F4, F8, and AF4 (Figure 1b).

### 2.3. Description of Classifiers

In this study, we used several classification algorithms: decision tree, support vector machine and k-nearest neighbor. DT [32,33,34] is a widely-used and easy-to-implement classification technique used to analyze data for prediction purposes. It consists of a set of conditions or rules organized in a hierarchical structure where the final decision can be determined following conditions established from the root to its leaves. SVM [35,36,37] refers to supervised learning models used to classify data into two categories by finding an optimal hyperplane that separates two possible values for the variable y∈ {+1, −1}. If the data can be separated linearly, the hyperplanes divide SVM input data into two initial subgroups by assigning {−1, +1} tags. KNN [38] is a non-parametric approach used to solve classification and regression problems, based on the assumption that an object’s class is the same as that of its closest neighbors.

Both decision trees and KNN are designed to handle multi-class problems, which is not the case for the SVM classifier as this is an algorithm originally developed for use with two classes only. However, the classification can be expanded to more than two classes by combining binary classifiers, that is, by generating a classifier for the *d* classes available. For instance, it could be used thus: classifier 1 (class 0 *vs.* class 1 and class 2), classifier 2 (class 1 *vs.* class 0 and class 2), classifier 3 (class 2 *vs.* class 0 and class 1). A decision function is used subsequently to group them and assess the class they belong to following this criterion: class 0 (1 0 0), class 1 (0 1 0) and class 2 (0 0 1).

## 3. Proposed Method

In signal-pattern recognition, each sample is represented by a collection of descriptors that will be segmented and classified. Thus, the characterization of signals, in this case EEG signals, is essential to determine the performance and accuracy of the final classification process. As mentioned in Section 1, the analysis in the frequency domain is a common technique used when extracting features of EEG signals; for instance, the Fourier transform, short Fourier transform or wavelet transform are commonly used. However, the use of quaternion algebra is proposed as a novel tool to extract EEG-signal features that simplifies the final classification task.

EEG signals can be described using quaternion-based rotations and orientations, as shown earlier. Vector rotations are usually described by the rotation matrix or Euler angles [39], and quaternions can be advantageous for three reasons: (1) they avoid ambiguities in the data; (2) they allow a more accurate representation of the data; and (3) they require fewer calculations than other rotational techniques.

The proposed so-called quaternion-based signal analysis (QSA) method described in Algorithm 1 (Table 2) can be used to model multichannel EEG signals using quaternions, taking a set of input signals as a single entity, and converting it into a pure quaternion.

Thus, the multichannel EEG information is represented using quaternions to characterize signals recorded for 10 min during each test, and classified after further processing subsequently.

Algorithm 1 can be described as follows: Line 1 defines the input data: delta (*dt*) movements in time *t*, signals to be analyzed (*signals*) and blocks with the channels to be analyzed (*nblocks*), where each block includes signals from four channels, and pr is a flag to indicate whether the sample is being used during validation or training. Line 2 calculates the segments matrix *y(t)* defined by changes between the three classes (left, right and waiting time) from the pool of signals contained in *nblocks*. The quaternions array *quat,* at line 3, is created using the channels included in *nblocks*. Line 4 dictates that for each segment *y_i_*, *q(t)* and *r(t)* are formed considering that *q(t)* is an array with *n* quaternions (line 4a) and *r(t)* is an array with *n* pure quaternions moved according to a *dt* value (line 4b). After this, the rotation *q_rot_(t)* is calculated using quaternions *q(t)* and *r(t)* (line 4c), which produces an array of *n* rotated quaternions. Line 4d calculates the scalar array *q_mod_(t)* from the module *q_rot_(t)*, which contains *n* scalar elements that will be used at line 4e to form a matrix with *M_i,j_* features, where index *i* corresponds to the analyzed segment and index *j* is one of the *m* features to be analyzed using the equations included in Table 3. Line 4f gives shape to vector *c_i_* using the classes assigned to each segment *y_i_.* In addition, matrix *M_k,j_* is created using k-th data for the training phase at a *%t* rate (line 5) and matrix *M_l,j_*, together with the remainder of the original matrix *M_ij_*, is used to create the validation where index *l* corresponds to the elements used in the validation process in a *1−%t* proportion to the analyzed segment (line 6). Thus, in lines 7 and 8 data are trained and/or validated by calling the function *ProcQSA()* and returning a vector R using classes ${\hat{C}}_{k}$ (class aimed for training purposes) and ${\hat{C}}_{l}$ (class aimed for validation purposes). In the function *ProcQSA()*, during the training phase, matrix *Mt* data are assessed using DT, KNN or SVM classifiers, adjusting the parameters for each classifier. Finally, the accuracy percentages are calculated during the training and validation phases (lines 9 and 10).

Table 3 shows the formulae used to extract features by Haralick [40] and adapted for use with quaternion algebra to implement the QSA model.

Equation (7) shows matrix M with its features vector. In this matrix columns correspond to features and rows to samples. The features vector consists of the average, variance, contrast and homogeneity.

(7) $$M=\left[\begin{array}{cc} \begin{array}{cc} \mu_{1} & \sigma_{1}^{2}\\ \mu_{2} & \sigma_{2}^{2} \end{array} & \begin{array}{cc} con_{1} & H_{1}\\ con_{2} & H_{2} \end{array}\\ \begin{array}{cc} \vdots & \vdots \\ \mu_{i} & \sigma_{i}^{2} \end{array} & \begin{array}{cc} \vdots & \vdots \\ con_{i} & H_{i} \end{array} \end{array}\right]$$

## 4. Description of the Experimental Tests

This section outlines the steps followed to implement the proposed QSA method (Algorithm 1) with EEG signals obtained from various participants for the purposes of detecting three mental states: thinking “left”, “right” and “waiting time”, with no need for any movement or word.

First of all, the technique consists in representing the EEG signals as a single quaternion. For experimental testing, one channel of the EEG signal is used as the real component and three more channels as the imaginary components. After that, feature computation provides the input data for the KNN, SVM and DT classifiers.

**Figure 2:**
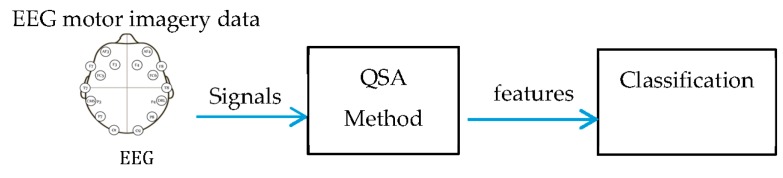
Block diagram of the overall EEG signal classification strategy.

EEG signals are acquired using the Emotiv Epoc headset (shown above in Figure 1a). The graphic user interface used to analyze the acquired signals is Python-based, and the feature selection and extraction is programmed using Matlab R2014a^®^. The following subsections include detailed descriptions of each stage of the block diagram (Figure 2).

### 4.1. EEG Signal Acquisition

**Figure 3:**
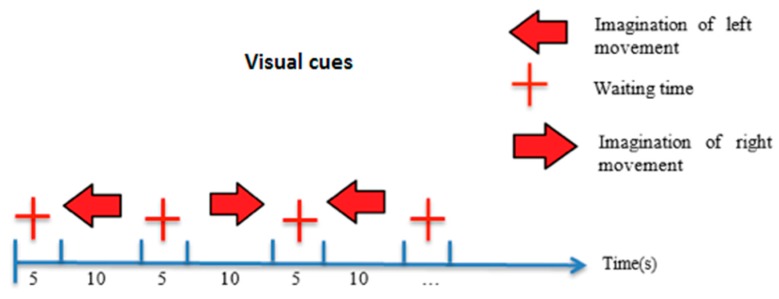
Visual cues with timing scheme.

For signal acquisition purposes, the BCI’s cognitive mode was used, given that motor activities were one of the cognitive processes involved in producing movement, which was the key focus of the tests. The experiment was conducted with 10 participants of several ages, both male and female. The time for each individual test was 10 consecutive minutes, during which three elements appeared on the screen: an arrow pointing to the left and moving in that direction, a cross representing rest time and an arrow pointing to the right and moving in that direction (see Figure 3). The left and right arrows alternate after appearing for 10 s (test mode) with a 5 s rest time in between during which a cross appeared at the center of the screen. In other words, a total of 40 arrows were shown alternately for 10 min, with 5 s breaks between each alternation. The purpose of the experiment was to make the participant think or imagine movement while the arrow appeared on the screen. In addition, during the test mode, participants were asked to remain motionless and avoid sharp body movements that could interfere with the signal being recorded, which they did, but were then allowed to move freely during rest time. States 0, 1 and 2 were used to refer to the three elements: waiting time (0), left (1) and right (2) during the classification phase.

The number of samples recorded during the EEG data acquisition phase from each of the 10 participants amounts to 76,800, considering eight of the 14 available channels on the Emotiv Epoc device in relation to activity in the motor and frontal areas of the brain. Later, the samples were combined (Table 4) to form signal blocks (*nblocks*) that were then used as input in Algorithm 1 in order to find the channel with the best performance.

**Figure 4:**
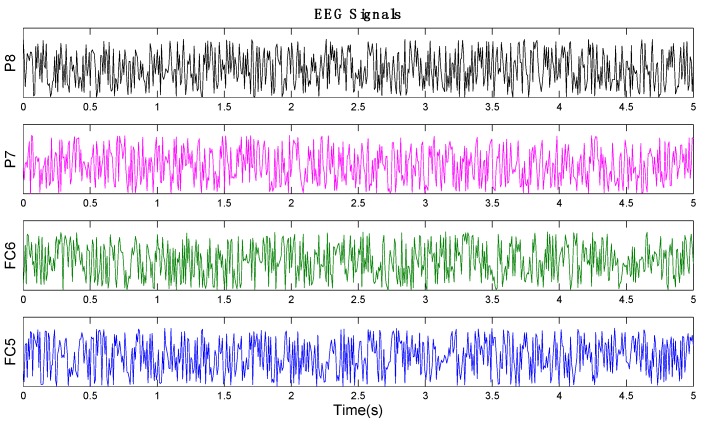
Example of block 1 EEG signals from four channels captured by the Emotiv Epoc device for 5 s.

Figure 4 shows the signals acquired from block 1 for 5 s.

### 4.2. QSA Method Implementation

**Figure 5:**
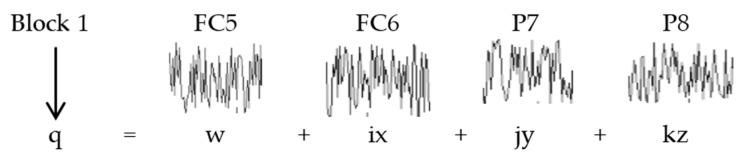
Creating the quaternion using elements of block 1, with FC5 channel as the scalar component and FC6, P7 and P8 as the imaginary components.

After acquiring the data in the previous step, the next phase consisted of extracting EEG signal features to find the desired classes (thinking “left”, “right” and “waiting time”). First, the QSA method was implemented (Algorithm 1) to represent EEG signal blocks as shown in Table 4, and to extract the features related to the cues shown. In these experiments pre-processing was not required. Thus, to detect sharp changes between classes a segments matrix *y(t)* was obtained using 1200 samples for each 10 s acquired for “left” and “right” classes, and 600 samples for the “waiting time” class obtained for each 5 s-long breaks. In addition, the quaternion (*quat*) emerged from block signals considering the first channel as the scalar component and the remaining ones as the imaginary components, as shown in Figure 5. Later, *q(t)* and *r(t)* were calculated, the latter with its relevant *dt* movement. As for the movement, several tests were conducted using different *dt* values for each block. In other words, a *dt* movement of 1 to 10 was used in multiples of 7.8 ms (sample period) at point in time *t* to find out its impact on the classification accuracy percentage. Rotation *q_rot_* was calculated using *q(t)* and *r(t)*, as well as *q_mod_* using the result of rotation *q_rot_*. Once the module was obtained, the four features included in Table 3 (Mean, Contrast, Homogeneity and Variance) were calculated to generate matrix *M_i,j_* and vector *c_i_* (where *i* represents the segments of *y(t)* and *j* represents the features). The resulting matrix *M_i,j_* was using during the training and classification process to infer the mental state and the required class.

### 4.3. Classification

During the training and validation stages, several samples were taken to create submatrix *M_k,j_* using 30% of *M_i,j_* on condition that the data contained examples of the three classes required, that is, “left” (1), “right” (2) and “waiting time” (0). In addition, to create matrix *M_m,n_* during the piloting phase, 70% of the data were used. In this study, the training and validation process was conducted using three classifiers: (1) decision tree based on input and predictor variables; (2) SVM using a kernel-type Gaussian RBF with a default scaling factor; and (3) KNN, which uses the Euclidean distance with a number of default Matlab-selected neighbors. The classification process was repeated 20 times for each of the 10 movements mentioned in each block. The methodology was applied to each of the 10 participants with the following results: 10 participants × 10 blocks × 10 *dt* × 20 repeats.

Considering all these parameters, a detailed analysis of the performance and best accuracy for each classifier for various signal blocks and movements is presented in the following sections.

## 5. Results and Discussion

The accuracy percentages obtained during the classification phase are shown in Table 5. The table presents the accuracy rate for the three classifiers for each *dt* movement. As can be seen, with a movement *dt =* 4, DT is the classifier with the best classification percentages at 84.92%, followed by KNN at 84.34%. In contrast, when the movement was *dt =* 2, KNN was 84.39%. Finally, SVM recorded the lowest classification percentages, such as 77.49% for *dt =* 4.

**Figure 6:**
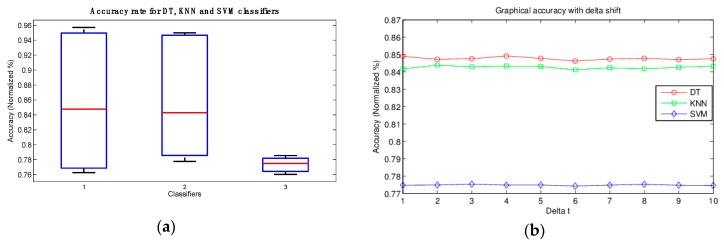
Graphical representation of accuracy rates: (**a**) for DT (1), KNN (2) and SVM (3); and (**b**) accuracy with delta movement.

Figure 6, in turn, shows the graphs that correspond to Table 5 data. Figure 6a shows the symmetry of data for the three classifiers. In addition, in Figure 6, both (a) and (b) show that data for DT and KNN are both close to 84%, while the SVM mean was recorded as 78%.

On the other hand, Figure 6b shows the accuracy of the various classifiers. Note that parameter “*dt*” does not alter the accuracy of the results and as a result does not affect the QSA model.

Table 6 shows the signal block performance results for the 10 blocks under analysis. Block 1 had the best results for DT and KNN with an accuracy in excess of 86% for both classifiers at movements of 1 and 3. As for SVM, it achieved an accuracy rate of 78% in block 4 with a movement *dt* = 8.

Table 7 shows that block 1 achieved the best average results for DT and KNN, unlike SVM which performed at its best in block 3.

**Figure 7:**
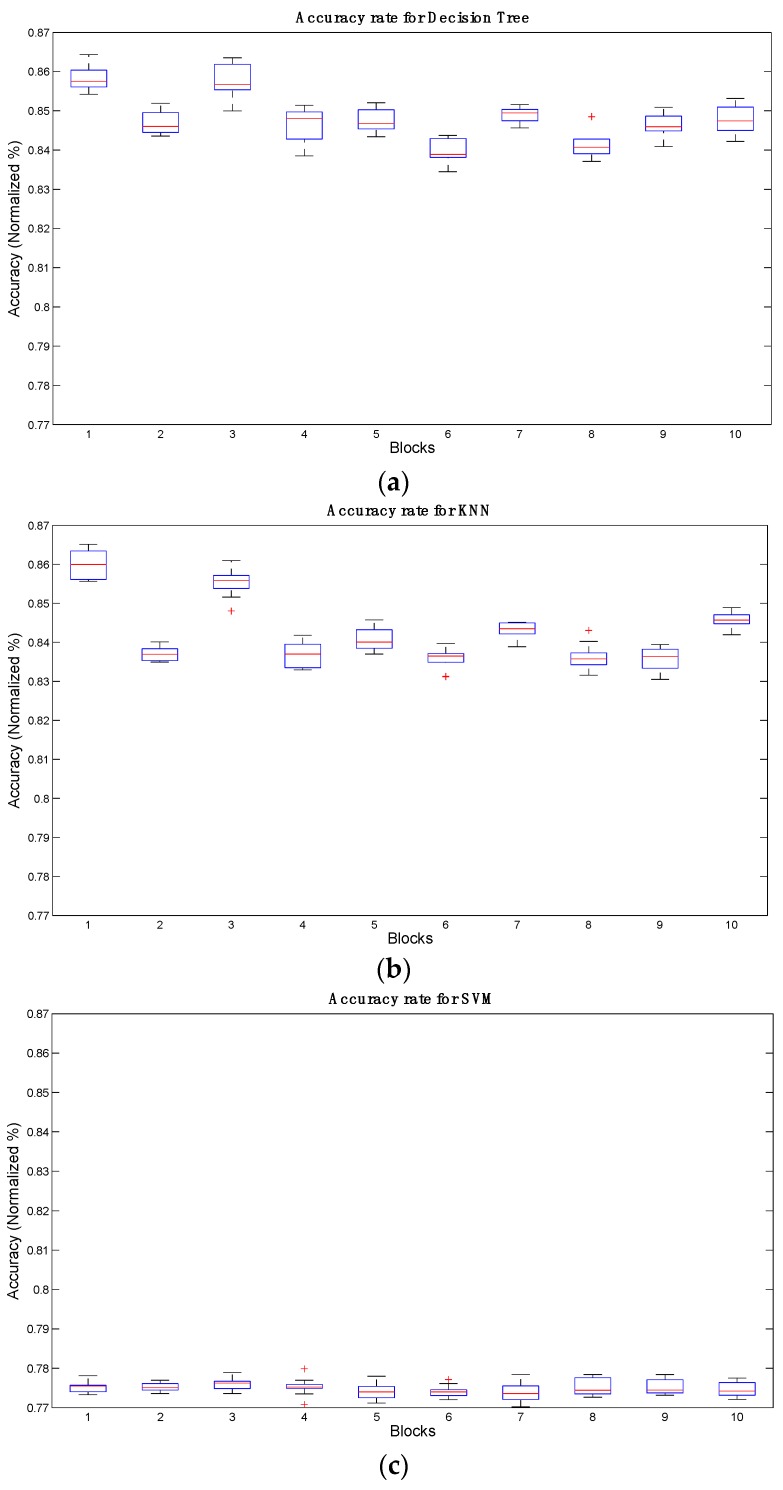
Graphical representation of accuracy rates for: (**a**) DT; (**b**) KNN and (**c**) SVM for different blocks.

Figure 7 shows the behavior of blocks by classifier. As can be seen, blocks 1 and 3 stand out for all three classifiers. In contrast, block 6 shows a poor performance for DT at 84.44%, block 9 produced poor results for KNN at 83.04% and block 7 did likewise for SVM at 77.02%.

**Figure 8:**
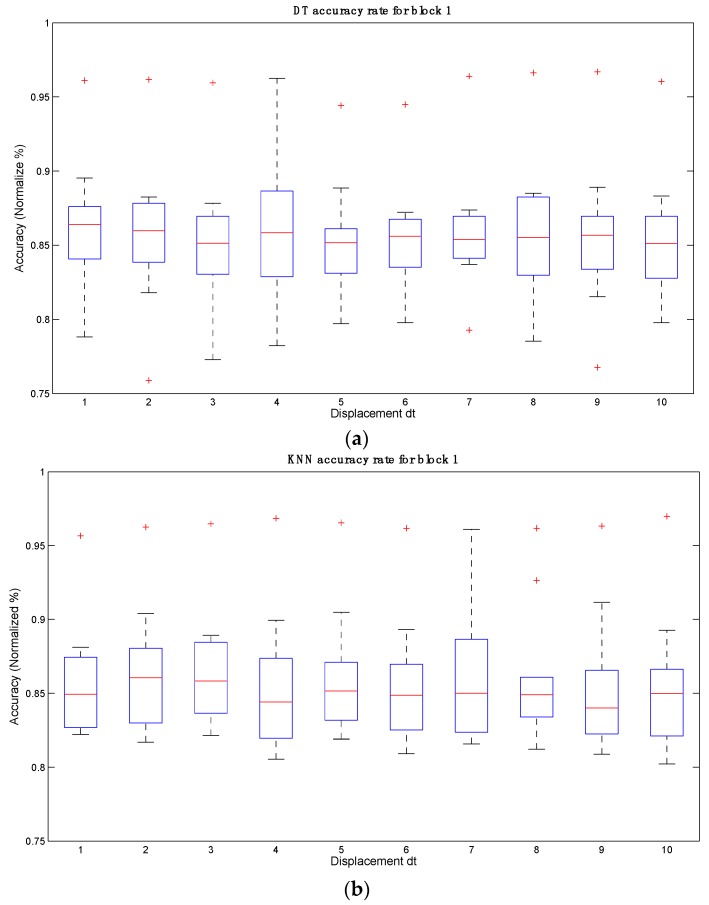

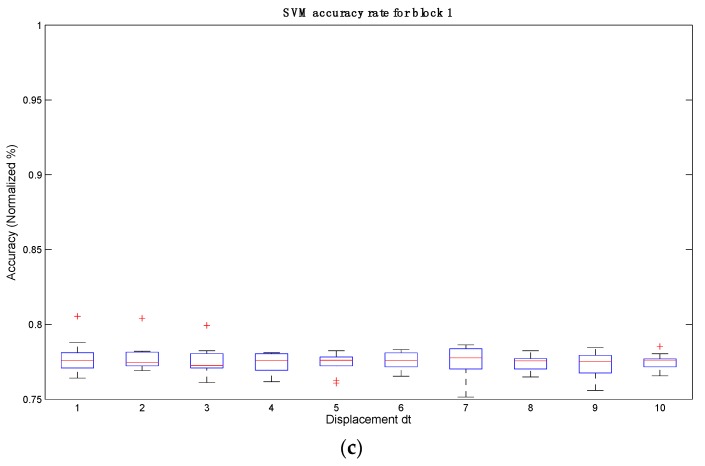
Graphical representation of accuracy rates for block 1 using: (**a**) DT; (**b**) KNN and (**c**) SVM for block 1 and *dt* = 4.

Therefore, the best accuracy results were obtained by block 1 with a movement *dt* = 4. Table 8 and Figure 8 show the behavior of data obtained for each participant in block 1.

Subject 1 consistently achieved the best results compared to the remaining participants because this was the only participant that was trained using the BCI device and data-acquisition system. However, considering that the remaining participants had no training, their results were good nonetheless.

The performance obtained by the three classifiers has been compared using various assessment metrics, such as recognition rate (RT) and error rate (ET). However, according to Ibarra [41], these metrics do not always suffice to assess a classification method. For instance, in some cases the estimate of the recognition rate may not contain enough information to be assessed. Therefore, further metrics ought to be used, such as sensitivity (S_d_) and specificity (Sp_d_), and four complementary performance metrics: accuracy (A_d_), false alarm rate (FA_d_), positive probability (PP_d_) and negative probability (NP_d_), where subscript *d* corresponds to the class in point.

The sensitivity metric assesses how aptly the classifier can recognize samples from the class in point. Specificity, also known as real negative rate, measures whether the classifier can recognize samples that do not belong to the class in point. Accuracy assesses the number of samples classified within the class in point that actually belong to it. The false alarm rate records fake positives or type-I errors. Positive probability is concerned with the proportion of samples within the class in point that has been classified correctly compared to the samples that do not belong to the class in point and have been misclassified. This latter metric is weighted considering the number of samples in each class. Negative probability measures the proportion of samples classified as not belonging to the class in point. This metric is weighted considering the number of samples in each class.

(8) $$RT=\frac{\#\left\{c|c\;=\hat{c}\right\}}{\#\left\{c\right\}}$$

(9) $$ET=\frac{\#\left\{c|\;c\neq \hat{c}\right\}}{\#\left\{c\right\}}$$

(10) $$S_{d}=\frac{\#\left\{c|\;c=d\;\&\;c=\hat{c}\right\}}{\#\left\{c|\;c=d\;\right\}}$$

(11) $$Sp_{d}=\frac{\#\left\{c|c\;\neq d\;\&\;c=\hat{c}\right\}}{\#\left\{c|c\;\neq d\;\right\}}$$

(12) $$A_{d}=\frac{\#\left\{c|\;c=d\;\&\;c=\hat{c}\right\}}{\#\left\{c|\hat{c}\;=d\;\right\}}$$

(13) $$FA_{d}=\frac{\#\left\{c|c\;\neq d\;\&\;c\neq \hat{c}\right\}}{\#\left\{c|c\;\neq d\;\right\}}$$

(14) $$PP_{d}=\frac{S_{d}}{1-Sp_{d}}$$

(15) $$NP_{d}=\frac{1-S_{d}}{Sp_{d}}$$

The results are presented in Table 9, highlighting the best score delivered by each performance measure using the three classifiers.

Note that in Table 9 the DT classifier shows the best performance for the recognition, sensitivity, specificity, accuracy and positive probability rates. The results for KNN are similar to those for DT, but the former shows a better accuracy performance with class 0. The SVM classifier shows the worst performance in recognition and error, but has a better performance in sensitivity with class 1 and accuracy for class 0. The error rates are 15.25% for DT, 16.38% for KNN, and 22.65% for SVM, all of them still within an acceptable range.

To sumrize, the results of testing this new method with 10 participants 20 times per participant using 30%–70% of the data have been presented. The average performance accuracy was 84.75% when using the DT classifier. The same test was performed using DT and SVM classifiers that yielded an accuracy rate of 83.62% for KNN and 77.35% for the SVM classifier. After exhaustive tests of the proposed technique, the results show that this methodology for monitoring, representing and classifying EEG signals can be usefully applied for the purposes of having individuals control external devices.

## 6. Conclusions

Representing rotations in three-dimensional spaces using quaternions is computationally more efficient, both in terms of storage space required and the number of necessary operations. Thus, the results presented in this article show that QSA can be used as a tool to extract EEG signal features to identify “left” and “right” accurately using DT and KNN classifiers, even though admittedly the tests were conducted in a semi-controlled environment.

This study has presented the QSA method to obtain features of EEG signals and to represent motor instructions. The differences between these features were assessed using DT, SVM and KNN classifiers. The best classification results were obtained using DT. The QSA technique opens up new modeling and classification opportunities to process both biosignals and other types of signals for BCI devices. The QSA technique requires greater levels of independence between signals embedded into each quaternion for best results. In the future, this technique should be modified to reduce the number of samples needed to obtain a class and thus the analysis and processing times. In addition, the number of cognitive classes should be increased to include facial and emotional expressions in tests as well.

## Figures and Tables

**Table 1:** Operations using quaternions *q* and *p*.

Operation	Formulae
Addition	$q+p=\left(s_{q}+s_{p},\;v_{q}+v_{p}\right)$
Multiplication	$q\;⨂\;p=\;\left(s_{q}s_{p}-\;v_{q}\cdot v_{p},\;s_{p}v_{q}+s_{q}v_{p}+\;v_{q}\times v_{p}\right)$
Scalar product	$q\cdot p=\left(s_{q}s_{p},\;v_{q}\cdot v_{p}\right)$
Conjugate	$\bar{q}=\left(s_{q},\;-v_{q}\right)$
Norm	$\|q\|=\sqrt{\bar{q}⨂q}=\;\sqrt{q⨂\bar{q}}=\;\sqrt{s_{q}^{2}+{\left\|v_{q}\right\|}^{2}}$
Inverse	$q^{-1}=\frac{\bar{q}}{{\left\|q\right\|}^{2}}$

**Table 2:** QSA Method for training and classifying EEG signals.

Algorithm 1
Inputs: dt, signals, nblocks, pr y(t) ← segments of signals quat ← signals (nblocks) For each y_i_(t) do q(t) ← quat(t) r(t) ← quat(t-dt) q_rot_(t) ← n_rot_ (q(t), r(t)) q_mod_(t) ← *mod*(q_rot_(t)) *M_i,j_* ← f*_j_ (q_mod_(t))* *{j = 1,…, m}* c_i_ ← *{ c=(1,2,3,...,n)|y_i_(t)* ∈ *c}*
End for 5. *M_k,j_* ←{ *M_i,j_* |$\frac{\#k}{\#i}=\%t$ } 6. *M_l,j_* ← *{ M_i,j_ |{l}* *℘* *{k} ,*$\frac{\#l}{\#i}=1-\%t$*}* 7. [${\hat{C}}_{k}$, pr] = ProcQSA( pr, *M_k,j_*, c_k_) 8. [${\hat{C}}_{l}$, pr] = ProcQSA(pr, *M_l,j_*, c_l_) 9. %rt = $\frac{\#\{C_{k}\;|C_{k}=\;{\hat{C}}_{k}\}}{\#\left\{C_{k}\right\}}$ 10. %rv = $\frac{\#\{C_{l}\;|C_{l}=\;{\hat{C}}_{l}\}}{\#\left\{C_{l}\right\}}$ Function ProcQSA(pr, Mt, c) if pr == true then a. R = training(Mt,c) b. pr = false else b. R = classify(Mt) end if return [R,pr]

**Table 3:** Statistical features extracted using quaternions.

Statistical Features	Equation
Mean (μ)	$=\frac{\Sigma \left(\;q_{mod}\right)}{N}$
Variance $\left(\sigma^{2}\right)$	$=\;\frac{{\left(\sum^{\text{​}}{\left(q_{mod}\right)}^{2}-\mu \right)}^{2}+\sum^{\text{​}}{\left(q_{mod}\right)}^{2}}{2N}$
Contrast (con)	$={\frac{\Sigma \left(\;q_{mod}\right)}{N}}^{2}$
Homogeneity (H)	$=\sum^{\text{​}}\frac{1}{1+{\left(q_{mod}\right)}^{2}}$
Cluster Shade (cs)	=$\sum^{\text{​}}{\left(q_{mod}-\mu \right)}^{3}$
Cluster prominence (cp)	=$\sum^{\text{​}}{\left(q_{mod}-\mu \right)}^{4}$

**Table 4:** Signal blocks.

Block	BCI Channel			
1	FC5	FC6	P7	P8
2	FC5	FC6	T7	T8
3	FC6	FC5	P7	P8
4	FC6	FC5	T7	T8
5	F3	F4	FC5	FC6
6	F3	F4	FC5	FC6
7	F4	F3	FC5	FC6
8	F4	F3	T7	T8
9	T7	T8	FC5	FC6
10	T7	T8	P7	P8

**Table 5:** Accuracy rate for DT, KNN and SVM classifiers with *dt* movement.

*dt*	Classification Accuracy								
	DT			KNN			SVM		
	MAX	MEAN	MIN	MAX	MEAN	MIN	MAX	MEAN	MIN
1	0.9572	0.8490	0.7662	0.9440	0.8418	0.7857	0.7815	0.7748	0.0000
2	0.9551	0.8473	0.7633	0.9490	0.8439	0.7852	0.7843	0.7749	0.0000
3	0.9471	0.8476	0.7660	0.9474	0.8429	0.7851	0.7837	0.7754	0.7662
4	0.9507	0.8492	0.7686	0.9487	0.8434	0.7826	0.7828	0.7749	0.7633
5	0.9516	0.8478	0.7628	0.9468	0.8432	0.7836	0.7822	0.7750	0.7660
6	0.9468	0.8463	0.7658	0.9457	0.8412	0.7777	0.7809	0.7743	0.7686
7	0.9534	0.8474	0.7638	0.9484	0.8424	0.7876	0.7836	0.7749	0.7628
8	0.9495	0.8478	0.7760	0.9473	0.8418	0.7837	0.7820	0.7753	0.7658
9	0.9504	0.8471	0.7722	0.9499	0.8427	0.7849	0.7855	0.7748	0.7638
10	0.9568	0.8475	0.7649	0.9490	0.8433	0.7879	0.7828	0.7747	0.7753

**Table 6:** Best accuracy rates for signal blocks using classifiers DT, KNN and SVM.

Classifier	Signal Blocks									
	1	2	3	4	5	6	7	8	9	10
**DT**	**0.8644**	0.8519	0.8635	0.8514	0.8520	0.8437	0.8516	0.8485	0.8509	0.8531
**KNN**	**0.8651**	0.8101	0.8609	0.8418	0.8457	0.8396	0.8451	0.8431	0.8394	0.8490
**SVM**	0.7781	0.7770	0.7790	**0.7799**	0.7780	0.7774	0.7784	0.7784	0.7784	0.7775

**Table 7:** Average accuracy rate for signal blocks using classifiers DT, KNN and SVM.

Classifier	Signal Blocks									
	1	2	3	4	5	6	7	8	9	10
**DT**	**0.8584**	0.8472	0.8575	0.8467	0.8477	0.8398	0.8489	0.8417	0.8463	0.8478
**KNN**	**0.8599**	0.8370	0.8553	0.8371	0.8408	0.8357	0.8432	0.8363	0.8357	0.8457
**SVM**	0.7752	0.7752	0.7760	0.7753	0.7741	0.7741	0.7739	0.7739	0.7754	0.7746

**Table 8:** Accuracy rate for 10 subjects using classifiers DT, KNN and SVM (block 1 and *dt* = 4).

Classifier	Subjects									
	1	2	3	4	5	6	7	8	9	10
**DT**	0.9427	0.8425	0.8324	0.8291	0.8662	0.8672	0.8649	0.8098	0.7632	0.8574
**KNN**	0.9471	0.8313	0.7946	0.8122	0.8534	0.8508	0.8642	0.7875	0.7735	0.8473
**SVM**	0.7625	0.7702	0.7710	0.7797	0.7811	0.7687	0.7818	0.7788	0.7581	0.7831

**Table 9:** Comparison of eight performance measures for the three classifiers using signal block 1, for *dt* = 4. The best performance results are highlighted in bold.

Performance Measures	DT	KNN	SVM
RT	**0.8475**	0.8362	0.7735
ET	**0.1525**	0.1638	0.2265
S_0_	**1**	1	1
S_1_	0.6505	0.6344	**0.8775**
S_2_	**0.6701**	0.6349	0.0938
Sp_0_	**0.6598**	0.6343	0.4948
Sp_1_	**0.9064**	0.8964	0.7427
Sp_2_	**0.8972**	0.8926	0.9638
A_0_	0.9978	**0.9979**	**0.9979**
A_1_	**0.6944**	0.6548	0.6317
A_2_	**0.6663**	0.5002	0.1404
FA_0_	0.3402	0.3657	**0.5052**
FA_1_	0.0936	0.1036	**0.2573**
FA_2_	0.1028	**0.1074**	0.0362
PP_0_	**4.1122**	3.5360	1.9911
PP_1_	**8.3456**	6.6754	3.3885
PP_2_	8.6331	8.4926	**0.9183**
NP_0_	0	0	0
NP_1_	0.3830	**0.4094**	0.1524
NP_2_	0.3647	**0.4107**	0.9343

## Abbreviations

The following abbreviations are used in this manuscript:

EEG	Electroencephalography
QSA	Quaternion-based signal analysis
BCI	Brain computer interface
DT	Decision tree
KNN	K-nearest neighbor
SVM	Support vector machine
FFT	Fast Fourier transform
PSD	Power spectral density
DWT	Discrete wavelet transform
CSP	Common spatial patterns
QFT	Quaternion Fourier transform
QPCA	Quaternion principle component analysis
RBF	Radial basis function
RT	Recognition rate
ET	Error rate
S	Sensivity
Sp	Specificity
A	Accuracy
PP	Positive probability
NP	Negative probability
FA	False alarm
QDA	Quadratic discriminant analysis
LDA	Linear discriminant analysis
MSE	Multi-scale entropy
MMSE	Multivariate multi-scale entropy
